# The relevance of pharmacological neuroenhancement for stress and resilience—A multistudy report

**DOI:** 10.3389/fpubh.2022.971308

**Published:** 2022-11-11

**Authors:** Julia Darwig, Petra Maria Gaum, Roman Pauli, Lina Nassri, Jessica Lang

**Affiliations:** Teaching and Research Area for Occupational Health Psychology, Institute for Occupational, Social and Environmental Medicine, RWTH Aachen University, Aachen, Germany

**Keywords:** pharmacological neuroenhancement, cognitive enhancement, substance misuse, stress, resilience, cross-lagged panel design, structural equation model – SEM

## Abstract

**Background:**

Pharmacological neuroenhancement (PNE) is discussed as coping strategy in academic and work-related contexts. Depending on the definition of PNE and sample population, different prevalence rates for various groups have been reported. In the three parts of the study, prevalence rates for work and student populations in Germany are detected and the reasons for PNE and possible causal associations between PNE, stress and resilience are investigated.

**Methods:**

In part 1 of the study, 152 occupational physicians (OPs) were surveyed about prevalence rates and reasons for PNE. In part 2 of the study, 1,077 German students reported on their PNE behavior. 704 students were then longitudinally considered to draw conclusions on causal associations between PNE, stress, and resilience.

**Results:**

The OPs' estimated prevalence rate of 10.9% in a working population is higher than the prevalence rate of 5.4% for prescription and illicit substances found in the student sample in part 2 of the study. The reason suspected by OPs to be most important for PNE with prescription drugs were performance pressure and long working hours. Using soft enhancers, such as caffeine, is most common with a prevalence rate of 76.8% in the student sample. Stress predicts a higher (β = 0.179, *p* < 0.001) and resilience a lower use of PNE (β = −0.13, *p* = 0.001). Resilience predicts a lower (β = −0.35, *p* < 0.001) and PNE a higher level of stress (β = 0.11, *p* < 0.001).

**Conclusion:**

OPs suspect a prevalence rate of 10.9% among the working population, while we found a prevalence rate of 5.4% among students. Caffeine is the most used substance for PNE, while the use of prescription and illicit substances remains low. Higher levels of stress and lower levels of resilience result in a higher use of PNE. Universities should therefore include the promotion of resilience and methods for dealing with study stress in health programs to reduce PNE.

## Introduction

Many students and employees experience stress due to performance pressure and a heavy workload (1, 2). Perceived stress, but also other intraindividual factors, may provoke individuals' maladaptive stress responses, including the risk for pharmacological neuroenhancement (3). Resilience, however, is a possible outcome when using adaptive coping styles and may mitigate the risk of PNE use (4). This study addresses all three topics: the prevalence of PNE in different populations, stress and resilience and possible causal associations between PNE, stress and resilience.

A growing importance of the discourse on PNE is observable, suggesting that PNE is an emerging phenomenon, especially in an academic context but also in work-related contexts (1, 5). PNE in a wider sense is defined as the use of psychoactive substances by healthy individuals for enhancing cognitive functions such as learning, concentration or mood (6). Some widely cited international authors focus on the use of prescription/illicit drugs for cognitive enhancement as the main substances for PNE (7, 8), but PNE can also include so called soft enhancers such as caffeine, e.g., in energy drinks (6). Maier and Schaub (9) claim that the reasons for substance abuse should always be considered in the respective PNE definition. Improving cognitive functions and compensating for stress are the most frequently reported reasons for PNE (9). Therefore, the general definition of PNE in the present study is regarded as the intake of soft enhancers and/or prescription/illicit substances for the reasons of improving cognitive functions such as concentration or vigilance and/or compensating for stress. The first part of this study is an expert's assessment of occupational physicians (OPs) investigating PNE in a working context. Since OPs are mainly confronted with PNE through prescription drugs due to their profession, PNE was considered to be taking prescription substances for performance enhancement in this part of the study [PNE through prescription drugs (PNE_PD_)].

A large span of lifetime prevalence rates between 1 and 20% have been reported for PNE, depending on sample population, definition of PNE, country, survey design and survey methods used (10–15). For example, PNE is not as widespread in Europe as in the United States (16). In 2005 and 2006, Teter et al. (17, 18) reported a lifetime prevalence rate of 8.1% for illicit use of prescription stimulants in an undergraduate student sample and 8.3% in college students in the United States, while Franke et al. (12) reported lifetime prevalence rates for cognitive enhancement with methylphenidate and amphetamines of 1.55% in German pupils and 0.78% in German students, respectively. Also, Sattler et al. (19) report a rather low prevalence rate of 3.2% in a sample of 5.882 German university students. However, one limitation of the latter study is that PNE assessment only included few substances; for example, the use of cannabis was not considered (19). Jebrini et al. (20) reported a prevalence rate of 8.8% for prescription drugs and 3.0% for illegal drugs in a sample of German medical students. The use of cannabis for PNE was not included in their definition of PNE as well. Comparably high prevalence rates to the findings of Jebrini et al. are reported for the working population: Müller et al. (21) reported a lifetime prevalence rate of 8.4% for non-medical use of prescription drugs in a sample with participants of four different occupations in Germany. Schröder et al. (22) report a similar prevalence rate (8.3%) in the German working population. Referring to the low last-year prevalence rate in comparison (2.8%), Schröder et al. (22) emphasize that what seems to be rather worrying is the overall disposition of workers to take psychoactive substances without medical indication (1, 22): in 2009, a survey of more than 3.000 German employees showed that 29.0% of the participants, whose work was mostly characterized by high stress, considered using PNE to improve memory and concentration at work to be acceptable (23).

Over time, different reasons for PNE use have been put forward. PNE is regarded as a dysfunctional coping strategy for stress because it is assumed that PNE conceals and not resolves stress and that PNE can be associated with health risks (24). Among students, coping with increased performance pressure and improving academic performance are the main reasons for PNE (9, 16, 23, 24). Middendorff et al. (2) published a survey among German students concerning their strategies for stress compensation and use of PNE. They found that 12% of participants had used PNE since the beginning of their studies to enhance their performance according to the requirements of their studies. Although the use of soft enhancers for PNE was queried in this study, some substances like coffee were not considered for PNE by Middendorff et al. (2). The same reasons for PNE use are prevalent the general working population, where perceived stress at work as well as general pressure to perform and long working hours or shift work are leading causes for PNE with prescription and illicit substances (9, 22, 23, 25, 26). A study by Chmitorz et al. (27) showed that spine surgeons with higher levels of stress seem to be more susceptible to PNE and that perceived stress is the main factor explaining the use of PNE. In a Swiss study, 34% of the investigated Swiss employees felt chronic stress. The authors estimated that 6% had made use of PNE with prescription drugs for improvement of cognitive functions in the past year and 15% had consumed substances to relax or sleep after a stressful working day in the last year (28). That PNE is used as a coping strategy for stress at work and university is also shown in a study by Dietz et al. (29): Stress and performance pressure lead to a 1.8-fold higher likelihood of PNE with prescription and illicit substances and a 10.2-fold higher likelihood of using soft enhancers. Also Jebrini et al. (20) report coping with stressful situations as the underlying motive for using PNE. Thus, there is an association between PNE and psychosocial working conditions, since PNE is one possible strategy to cope with work- or study-related stressors (1). Most often, stress is considered the predictor of PNE in cross-sectional studies, while at the same time there is also evidence that PNE might be the predictor for perceived stress. It is at least possible that the use of PNE might cause stress, anxiety, exhaustion and decreased performance, since little is known about the long-term results of PNE use (30).

Resilience is a possible outcome when using adaptive coping styles. Like PNE, concepts and definitions of resilience have changed over time, but still there is no consistent definition of resilience in scientific literature (31, 32). The reason for that is the lack of a common theory of resilience and an ongoing discussion on how resilience should be conceptualized and measured (33, 34). Still, most publications on resilience use the constructs of adversity and positive adaptation to stressors (35). Resilience is often referred to as a protective factor for stress and also defined as the “ability to recover from stress” (36, 37). Therefore, in the sense of the Resilience Theory resilience can be regarded as a key mechanism that protects against negative effects of unusually intense stress exposure and maladaptive coping styles such as PNE (32). Consequently, resilience should be regarded as a state and not a fixed character trait (38). In the present study, we integrate perceived stress and PNE into Resilience Theory. External stressors such as high performance pressure at work or in an academic context require adaptation (39). An individual who can adapt positively to such stressors and shows a stable trajectory of healthy functioning under these stressors can be considered resilient (32). Accordingly, resilience protects against maladaptive coping strategies such as the use of PNE (32, 39, 40).

Although resilience is an essential concept in adaptive stress-coping strategies, it has not been well documented in the literature on PNE to date (41). In 2018, Bagusat et al. (36) conducted a study among the general German population and found that participants who are less resilient had a higher risk of using PNE and that being more resilient decreased the risk of using stimulating and mood-modulating prescription drugs. In 2021, Jebrini et al. (20) showed that a higher PNE use in general, meaning soft enhancers and prescription/illicit substances, was associated with being less resilient in a sample of 1,159 medical students in Germany. These findings would fit into the theory that PNE can be considered a dysfunctional coping strategy under stress, whereas resilience would be associated with possessing more adaptive and positive coping strategies to encounter stress. In contrast to these findings, no statistically significant impact of psychological resilience or resilience factors on PNE use was found in the study of Chmitorz et al. (27). These differences might be explained by the different study samples and a different definition of PNE used in the respective studies: While Bagusat et al. (36) examined a sample from the general population and included the use of cannabis and additional stimulating illicit drugs in their definition of PNE, Chmitorz et al. (27) conducted their study among spine surgeons and used a narrower definition of PNE.

The concepts of perceived stress and resilience are strongly interrelated. Over the last years there has been a growing agreement that resilience should be conceptualized as the outcome of positive adaptation after exposure to stressors (42). This theoretical consideration is in line with study results by Tam et al. (43), who report that resilience mitigates the effects of perceived stress on PNE. Resilient people seem to grow in a positive direction after a time of high perceived stress rather than using maladaptive coping styles like PNE (44). Lupe et al. (45) even describe resilience as “the ultimate goal of stress management”, suggesting that building resilience may lead to less perceived stress. This is in line with the findings of Tam et al. (43), who showed that participants, who perceived a high level of stress, reported low levels of resilience.

Considering research on perceived stress, PNE and resilience, the aim of the present study is to gain more clarity about (a) frequency and (b) reasons for PNE use and (c) to investigate the causality between PNE, stress and resilience. For this purpose, at-risk populations namely the working population and students were analyzed. We followed our study aim with three different approaches to be able to draw a balanced picture of prevalence rates and causes of PNE and to get deeper insights about the time-lagged directed correlations between PNE, stress and resilience. First, we asked OPs as expert practitioners about their perception of the relevance of PNE and possible reasons for PNE use in the working population. This avoids biased answers from the population self-reports, because asking about the use of PNE can be sensitive and participants may not give correct answers. Hence, a social desirability effect may occur (46). Second, we asked university students directly about their PNE behavior, to test a more direct assessment of PNE prevalence rates with the focus of receiving reasons for PNE. Third, challenging Resilience Theory, we take the differential findings on the causal relationships between PNE, stress and resilience as an occasion to assess the use of PNE, stress and resilience in a longitudinal study design by following up on the student sample 1 month after initial assessment (*t*2). Hereby, we will get deeper insights in the time-lagged directed correlations between the variables and will be able to draw conclusions about possible causal associations between PNE, stress and resilience.

In sum, the following research questions (RQ) will be tested:

RQ1: What is the OPs perceived prevalence rate and what are perceived reasons for PNE_PD_ use among German employees (expert assessment)?

RQ2: What is the German students' prevalence rate of PNE and reasons or influencing factors for PNE usage (self-reported assessment)?

And finally, with the longitudinal student assessment the following hypotheses will be tested:

PNE and stress (model 1):

- Hypothesis 1 (H1): There is reversed causation between PNE and stress between time 1 (*t*1) and time 2 (*t*2).

PNE and resilience (model 2):

- Hypothesis 2 (H2): There is reversed causation between PNE and resilience between *t*1 and *t*2.

Resilience and stress (model 3):

- Research question 3 (RQ3): Is there a negative association between resilience at *t*1 and stress at *t*2?

## Methods

To achieve the above objectives of the study we have chosen different methodological approaches in the 3 parts of our study: Because self-reports on PNE may be biased due to social desirability (46), part 1 of our study asked OPs as experts to provide information on prevalence rates of PNE and reasons for PNE use to get an initial more objective hint regarding the relevance of PNE of its more extreme form in daily working life. OPs should not tend to answer socially desirable. At the same time, OPs estimate of prevalence rates are also imprecise, because they may focus on cases in need of treatment. Therefore, we conducted a direct assessment on self-reported PNE among students in part 2 of the study, looking at the origins of PNE in earlier stages of life. We reduced the social desirability bias as much as possible by conducting the survey using an anonymous online questionnaire. Thus, from the estimates of prevalence rates among OPs, which are accordingly probably somewhat overestimated, and the self-reported prevalence rates among students, which are accordingly probably somewhat underestimated, more light can be shed on what the order of magnitude of prevalence rates might be. For part 3 of our study 704 students that participated in part 2 were followed up longitudinally, to draw conclusions about causalities between PNE, stress and resilience.

### Procedure for RQ1

For part 1 of our study, we conducted an anonymous online questionnaire on OPs in Germany from May to November 2016. The OPs were approached *via* exclusive mailing lists of professional associations and social media.

Out of 458 clicks, 257 OPs started the online questionnaire and 163 reached the final page. Only finalized questionnaires were included. 5 OPs were excluded from the final sample, because they stated that they were not confronted with PNE_PD_ at work. 6 OPs were excluded, because they do not see work related factors as causes for PNE_PD_ and therefore did not provide the answers to the relevant scales of the study. The final sample for part 1 of our study consisted of 152 OPs, including 74 men (48.7%) and 78 women (51.3%). The mean age was 51.9 years (SD = 8.3).

#### Measures for part 1

Firstly, we ask OPs to indicate what percentage of employees they care for use PNE_PD_ on a regular basis. We then assessed how often employees ask OPs about possibilities for PNE_PD_ use to maintain or enhance performance, improve mood or reduce anxiety and agitation on a five—point Likert scale (1 = ‘never' to 5 = “always”). OPs perception of the increase or decrease in PNE_PD_ use over the past year was rated on a five—point Likert scale (1 = “strongly decreasing” to 5 = “strongly increasing”) and their perceived need for employees' education on risks and benefits of PNE_PD_ was rated on a four—point Likert scale (1 = “absolutely not necessary” to 4 = “absolutely necessary”). Finally, suspected work-related reasons for the use of PNE_PD_ were rated on a four—point Likert scale (1 = “completely unimportant” to 4 = “very important”).

#### Analytical strategy for part 1

All analyses for part 1 were performed using SPSS 25 (47). Descriptive statistics were used to evaluate perceived prevalence rates, the increase and decrease of PNE_PD_ and the reasons for PNE_PD_.

### Procedure for RQ2

For part 2 of our study, the data was collected using a self-designed survey and standardized questionnaires. The survey was presented as an online questionnaire in SoSci—Survey to ensure privacy and anonymity. The survey period was from 21.01.2020 to 18.03.2020. The survey languages were German and English. To recruit students, flyers with a QR code to access the survey were distributed in and in front of university buildings. Furthermore, the link to the survey was distributed *via* social media and sent to the student councils of different universities with the request to forward it to their respective students.

Out of 2,583 clicks, 1,205 participants started the online questionnaire and 1,101 reached the final page. Participants with missing values in key variables (PNE, semester, preparation for exams, field of study and gender) were excluded (*n* = 113). Additionally, participants who studied outside of Germany (*n* =5), were PhD students (*n* = 4) and had already finished their studies (*n* = 2) were excluded from further analyses. The final sample for part 2 of our study consisted of 1,077 participants, including 402 men (37.3%) and 675 women (62.7%). The average age was 24.0 years (SD = 3.8). Students from 84 different universities and all German states except Saarland participated. The vast majority (76.7%) came from North Rhine Westphalia and only one participant each from Bremen and Saxony-Anhalt.

#### Measures for part 2

**Figure 1:**
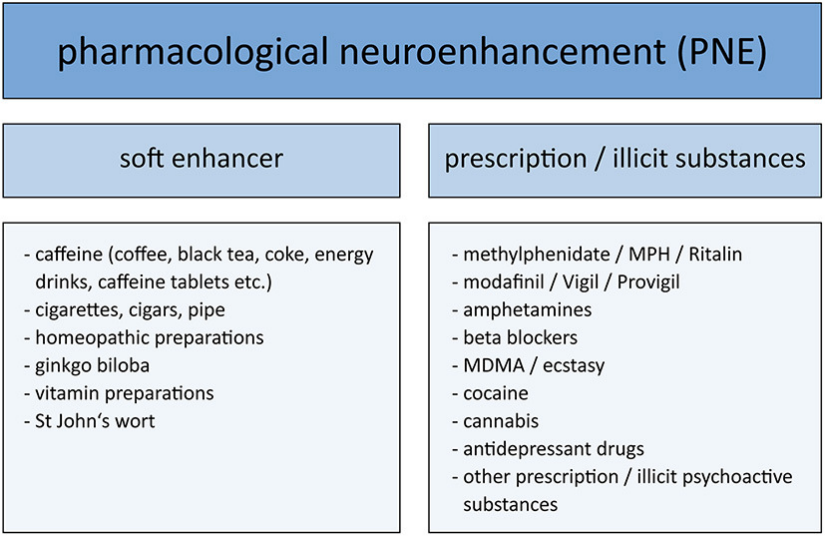
Substances used for pharmacological neuroenhancement surveyed in this study.

Different variables were collected. Firstly, the participants were asked if, in their opinion, prescription or illicit substances are taken without medical indication by some people to improve their cognitive functions, independently of their own use (“In your opinion: are illegal or prescription substances without medical indication, i.e., without medical necessity, taken by some people in order to improve their cognitive functions (“brain doping”)?”). This question was included in the questionnaire to evaluate overall disposition toward PNE under exclusion of the social desirability effect (46). Concerning their own use of PNE, participants were asked to indicate the frequency of the intake of soft enhancers, prescription drugs without medical indication and illicit drugs. For the individual substances please refer to Figure 1. Free space enabled participants to add specific substances.

To assess the prevalence rates of soft enhancers, students were first asked whether they used the respective substance “Rarely or never”, “1x per month”, “2–3x per month”, “1x to several times per week” or “daily” in the last month (e.g., “How often do you consume coffee/black tea/coke/energy drinks/other beverages containing caffeine […]?”). If they ticked “daily”, they were asked in a second step whether they had used the substances “1x”, “2x”, “3x”, “4x”, or “5x or more often” a day (e.g., “How often have you consumed one unit of the following beverages (i.e., one cup/one glass) or caffeine tablets (1 tablet) per day on average in the last month?”). Due to the lower prevalence rates of prescription and illicit substances, which can be assumed from the literature, the students were asked whether they had used these substances “never”, “1x”, “2x”, “3x”, or “4x or more often” in the last month (e.g., “In the last 4 weeks, how often have you taken any of the following substances without medical indication, i.e., without medical necessity?”).

The questionnaire then assessed the frequency of the reasons “compensation for study stress” and “improvement of cognitive functions” for the use of PNE and whether the substances taken had achieved the desired effect (e.g., “How often have you consumed one of these substances in the last month with the aim of compensating for study stress or improving your cognitive functions?”) [adapted from (2)]. To gain insight into the reasons for using PNE, students were asked how often (“Rarely or never”, “1x per month”, “2–3x per month”, “1x to several times per week” or “daily”) they had used the respective substance in the last month for improvement of cognitive functions. In a second step, they were asked how often they had used the substances in the last month for compensation of study stress.

#### Analytical strategy for part 2

All analyses for part 2 were performed using SPSS 25 (47). We calculated groupwise prevalence rates according to the reason for the use of the substance. All prevalence rates are past-month prevalence rates. Mean values of the groups for exam preparation or no exam preparation, gender and semester were compared—using independent samples two-tailed *t*-tests. The significance level was set at *p*-value < 0.05.

### Procedure for hypotheses 1–2 and RQ3

For part 3 of the study 704 students that participated in part 2 of the study were longitudinally considered to draw conclusions on causal associations between PNE, stress, and resilience. The methods used at *t*1 are explained in the respective section of part 2 of the study (please refer to lines 209–215). For *t*2, participants were asked to provide an email address, which was stored separately from their other data and could not be linked to them. Data collection for *t*2 took place between 20.02.2020 and 20.04.2020.

For response statistics at *t*1 please refer to the method section of part 2 of the study (please refer to lines 216–224). For *t*2 754 participants started the questionnaire and 704 reached the final page. Only participants who had no missing values in the key variables (PNE, stress, resilience) at *t*1 and *t*2 were included. Therefore, 50 participants were excluded, resulting in a final sample of 704 students that completed *t*1 and *t*2, including 234 men (33.2%), 458 women (65.1%) and 1 person with diverse gender (0.1%) for part 3 of our study. The gender of 4 participants was unknown (0.7%). The average age was 24.1 years (SD = 4.0y).

#### Measures for hypotheses 1–2 and RQ3

PNE: To generate a variable for PNE for *t*1 and *t*2, the number of different soft enhancers and prescription/illicit substances taken at *t*1 and *t*2 was added. The sum was weighted differently depending on the substance, as the effort to obtain and the inhibition threshold of taking prescription/illicit substances is higher than for soft enhancers. The number of soft enhancers was weighted by one while prescription/illicit substances were multiplied by two. Only those substances were considered for which improvement of cognitive functions or compensation for study stress were given as the reasons for use. Therefore, the following formula results for the PNE variable calculation: PNE = ∑different soft enhancer over the past month + (∑different prescription/illicit substances over the past month ^*^ 2). The PNE variable was calculated for *t*1 and *t*2 separately.

Stress: Student stress was assessed with the Higher Education Stress Inventory (HESI) (48) with the subscales for “Worries about future endurance/competence” (e.g., “I am worried that I will not acquire all the knowledge needed for my future.”), “Non-supportive climate” (e.g., “The studies have created anonymity and isolation among the students.”), “Faculty shortcomings” (e.g., “The teachers often fail to clarify the aim of the studies.”), “workload” (e.g., “Studies control my life and I have little time for other activities.”) and “financial concerns” (e.g., “As a student, my financial situation is a worry.”). This questionnaire consists of 21 items rated on a four-point Likert scale (1 = totally disagree to 4 = totally agree) (48). A mean scale was formed from all items (Cronbach's alpha: *t*1 = 0.82; *t*2 = 0.84).

Resilience: In a review Windle et al. (49) analyzed 19 resilience measurement scales and found no current “gold standard”. Most resilience scales measure resources that can contribute to maintaining or regaining mental health. Only the Brief Resilience Scale measures resilience as the ability to recover from stress and therefore as a process and not a fixed character trait (42, 50, 51). This scale consists of six items (e.g., “I usually come through difficult times with little trouble”) rated on a five-point Likert scale (1 = strongly disagree to 5 = strongly agree) (37). A mean scale was formed from all items (Cronbach's alpha: *t*1 = 0.82; *t*2 = 0.82).

#### Analytical strategy for hypotheses 1–2 and RQ3

For part 3 of the study, descriptive statistics and analyses of the key variables were performed using SPSS 25 (47). To analyze the causality between PNE, stress and resilience postulated in the hypotheses cross-lagged-panel-designs (CLPD) were used. To test our hypotheses, we considered three models with CLPDs: model 1: PNE and stress for H1a/b; model 2: PNE and resilience for H2a/b; model 3: resilience and stress for H3. The cross-lagged panels were calculated using the package lavaan (52) for structural equation modeling (SEM) in R Studio version 1.3.959 (53). The significance level was set at *p*-value < 0.05. To avoid non-convergence due to overfitted models, we did not include cross-sectional correlations in the model. Since the estimation of CLPDs by SEMs are influenced by the items distribution, we tested the items for multivariate normal distribution using the package MVN (54). Items did not hold the assumption of multivariate normal distribution (Mardia's skewness χ^2^= 829.1, *p* < 0.001; Mardia's kurtosis χ^2^= 23.8, *p* < 0.001), therefore we used maximum likelihood estimation with Satorra-Bentler scaled X2-test statistic providing robust parameter estimations when distribution assumptions are violated (55).

## Results

### Results part 1

For part 1 of our study, OPs estimated the prevalence rate for regular use of PNE_PD_ among the employees in their care at 10.9% (SD = 10.6). Of the OPs, 80.9% stated that they are “never” or “rarely” asked by employees about possibilities for PNE_PD_. Only 13.3% said that they are asked “sometimes”, 1.7% “often” and 2% “always” (mean = 1.8, SD = 0.9). 1.3% did not answer the question.

Most of the OPs estimated that the use of PNE_PD_ was either “increasing” (62.5%) or even “strongly increasing” (11.8%). Only 23.7% assumed the PNE_PD_ use to be constant, 2.0% decreasing or strongly decreasing (mean = 3.8, SD = 0.7). Accordingly, 44.7% stated that employees' education on risks and benefits of PNE_PD_ was “absolutely necessary” and 40.8% stated that it is “necessary”. Only 14.5% thought that it is “rather not necessary” or “absolutely not necessary” (mean = 3.3, SD = 0.7).

**Figure 2:**
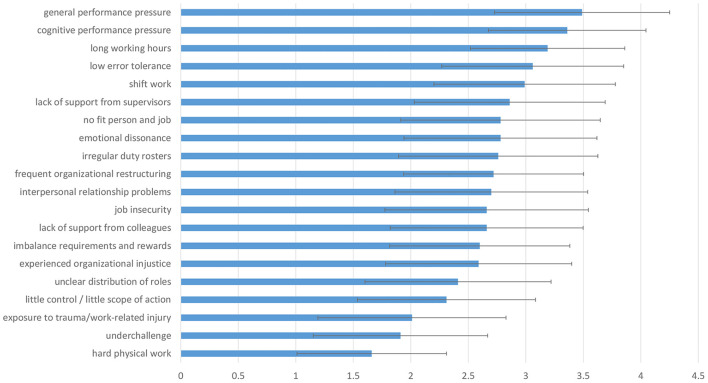
Suspected reasons for PNE_PD_ use among employees. It shows the mean and standard deviation of a four-point Likert scale (1, “completely unimportant” to 4, “very important”).

The reason suspected to be most important for PNE_PD_ intake among employees were general performance pressure, cognitive performance pressure and long working hours. Reasons of minor importance were e.g., personal conflicts among employees, unclear allocation of roles and hard physical labor. The frequencies of all mentioned reasons are illustrated in Figure 2.

### Results part 2

Results for part 2 of our study showed, that 83.1% of the responding students believed that prescription/illicit substances are used by students without medical indication to improve cognitive functions. Significantly more students (87.6%) who had not been preparing for exams in the last month believed that prescription/illicit substances are used without medical indication to improve cognitive functions than students who had been preparing for exams in the last month [81.9%; *t* (303.6) = 2.1, *p* = 0.04].

Regarding the prevalence rates of PNE for improvement of cognitive functions and compensation of study stress, 76.8% of respondents had made use of either soft enhancers or prescription/illicit substances for any of the two reasons within the past month. For both reasons, the prevalence rate for only prescription/illicit substances was lower with 5.4% within the past month. Students who had been in exam preparation for the last month showed a higher use of PNE (78.7%; for improvement of cognitive functions and/or compensation for study stress) than students who had not been in preparations for exams [66.7%; *t* (248.3) = 3.2, *p* < 0.001]. Table 1 contains further information on the prevalence rates for different groups and the different reasons for PNE.

**Table 1:** Pharmacological neuroenhancement by substance group and reason for use.

**Total**		**Improvement of cognitive functions**					**Compensation of study stress**		
	**Total**	**Soft enhancers**	**prescription/ illicit substances**	**Total**	**Soft enhancers**	**prescription/ illicit substances**	**Total**	**Soft enhancers**	**Prescription/ illicit substances**
	***n*** **(%)**	***n*** **(%)**	***n*** **(%)**	***n*** **(%)**	***n*** **(%)**	***n*** **(%)**	***n*** **(%)**	***n*** **(%)**	***n*** **(%)**
Total	827 (76.8%)	825 (76.6%)	58 (5.4%)	794 (73.7%)	794 (73.7%)	17 (1.6%)	583 (54.1%)	580 (53.9%)	49 (4.5%)
Gender									
Male	308 (76.6%)	307 (76.4%)	23 (5.7%)	303 (75.4%)	303 (75.4%)	6 (1.5%)	193 (48.0%)^a^	191 (47.5%)^a^	19 (4.7%)
Female	519 (76.9%)	518 (76.7%)	35 (5.2%)	491 (72.7%)	491 (72.7%)	11 (1.6%)	390 (57.8%)	389 (57.6%)	30 (4.4%)
Semester									
≤ 6	561 (76.3%)	559 (76.1%)	39 (5.3%)	539 (73.3%)	539 (73.3%)	12 (1.6%)	405 (55.1%)	403 (54.8%)	31 (4.2%)
>6	266 (77.8%)	266 (77.8%)	19 (5.6%)	255 (74.6%)	255 (74.6%)	5 (1.5%)	178 (52.0%)	177 (51.8%)	18 (5.3%)
Preparing for exams									
Yes	680 (78.7%)^b^	678 (78.5%)	50 (5.8%)	651 (75.3%)	651 (75.3%)	15 (1.7%)	484 (56.0%)^2^	481 (55.7%)^b^	41 (4.7%)
No	124 (66.7%)	124 (66.7%)	8 (4.3%)	123 (66.1%)	123 (66.1%)	2 (1.1%)	80 (43.0%)	80 (43.0%)	8 (4.3%)

Influencing factors considered are gender, semester and exam preparation.

*n*, number of participants in the considered group; %, relative frequency.

^a^Significantly different from female (independent sample two-tailed *t*-test, *p* < 0.05).

^b^Significantly different from no exam preparation (independent sample two-tailed *t*-test, *p* < 0.05).

The prevalence rates for prescription/illicit substances varied greatly by substance. Prescription/illicit substances were more often used for recreational purposes than for PNE. For information on substance use for other reasons than PNE please refer to Supplementary material 1. The prevalence rate was highest for the use of cannabis with a prevalence rate of 9.3% for the reason compensation of study stress only. The prevalence rates for other prescription and illicit substances were low and ranged between 0.5 and 1.7% for any reason. 16.4% (*n* = 177) of all students reported that they took a prescription/illicit substance in the last month for any reason, including recreational purposes. Of these, 27.7% (*n* = 49) consumed any substance to compensate for study stress and 9.6% consumed any substance for improving cognitive functions.

### Results part 3

**Figure 3:**
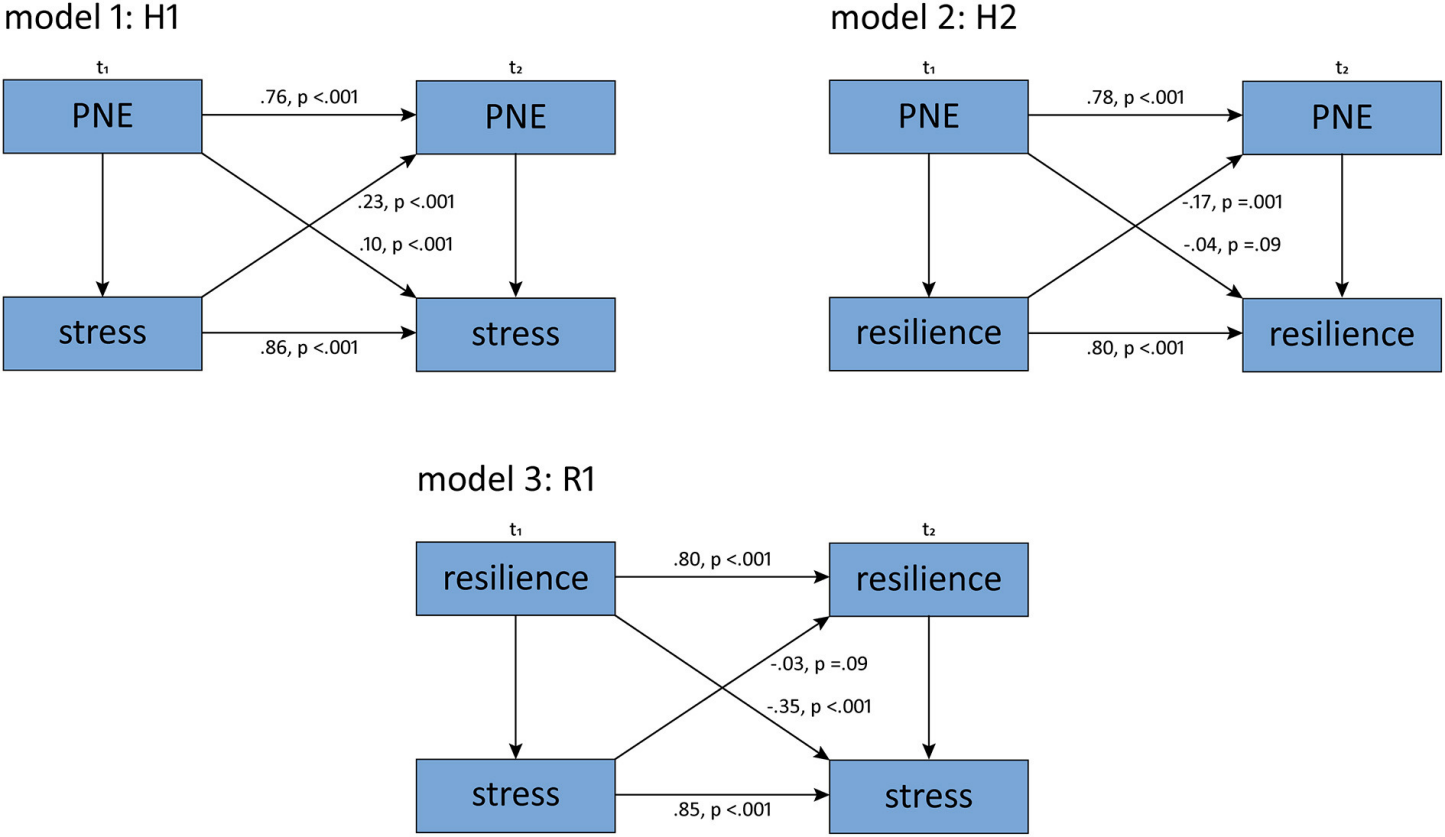
Standardized regression coefficients and *p*-values from the SEM for all three models. H1, hypothesis 1; H2, hypothesis 2; R1, research question 1; *t*1, time 1; *t*2, time 2; PNE, pharmacological neuroenhancement.

Part 3 of our study examined the causal relations between PNE, stress and resilience. Table 2 shows means, standard deviations and cross-sectional correlations for PNE, stress and resilience. Autoregressions and cross-sectional regressions were estimated by SEMs in the CLPDs and are shown in Figure 3. The fit indices for our models are shown in Table 3. Model fit was estimated using root mean squared error of approximation (RMSEA), standardized root mean square residual (SRMR), comparative fit index (CFI) and Tucker-Lewis Index (TLI) with regard to cut-off values as reported by Hu and Bentler (56): RMSEA ≤ 0.06, SRMR ≤ 0.08 and CFI and TLI ≥0.95. For model 1 and 2a good model fit can be assumed, since all fit indices were within the range suggested by Hu & Bentler (56). For model 3, the interference statistical goodness-of-fit criteria showed values above those suggested by Hu and Bentler, but the absolute and incremental fit measures showed good values. Accordingly, it can be assumed that although the model is only a moderately good approximation to the data, it explains the associations between the variables better than the baseline model.

**Table 2:** Descriptive parameters and correlation coefficients^a^ for the relevant variables.

		***t*** **1**			***t*** **2**		
		**M (SD)**	**PNE**	**Stress**	**Resilience**	**PNE**	**Stress**
	PNE	1.88 (1.77)					
*t*1	Stress	2.26 (0.42)	0.29^**^				
	Resilience	3.34 (0.79)	−0.16^**^	−0.37^**^			
	PNE	1.69 (1.63)	0.78^**^	0.23^**^	−0.17^**^		
*t*2	Stress	2.28 (0.42)	0.30^**^	0.86^**^	−0.35^**^	0.27^**^	
	Resilience	3.37 (0.74)	−0.16^**^	−0.35^**^	0.80^**^	−0.16^**^	−0.38^**^

M, mean; SD, standard deviation; PNE, pharmacological neuroenhancement; *t*1, time 1; *t*2, time 2 ^**^p < 0.01.

^a^Spearman's rho.

**Table 3:** Fit indices for model 1 (PNE and stress), model 2 (PNE and resilience) and model 3 (resilience and stress).

**Model**	**df**	**χ^2*a*^**	**Robust RMSEA**	**Robust RMSEA 90% CI**	**Robust SRMR**	**Robust CFI**	**Robust TLI**
Model 1	2	13.502	0.091	0.049–0.139	0.026	0.992	0.976
Model 2	2	0.230	0	0–0.037	0.004	1.000	1.005
Model 3	2	29.151	0.153	0.107–0.205	0.112	0.981	0.944

Df, degrees of freedom; RMSEA, robust root mean squared error of approximation; SRMR, robust standardized root mean square residual; CFI, robust comparative fit index; TLI, robust Tucker-Lewis-Index.

^a^Satorra-Bentler corrected.

In H1, the cross-lagged associations between PNE and stress were analyzed. Bivariate cross-sectional correlations between PNE and stress were significant at both measurement times (see Table 2). Likewise, the autocorrelations over time were significant for both variables, indicating that the constructs were stable over the time period (see Figure 3). The regressions over time show that PNE at *t*1 is a significant positive predictor of stress at *t*2 and that stress at *t*1 is a significant positive predictor of PNE at *t*2 (see Figure 3). Therefore, hypothesis 1 is accepted.

In H2, the cross-lagged associations between PNE and resilience were analyzed. Bivariate cross-sectional correlations between PNE and resilience were significant at both measurement times (see Table 2). Likewise, the autocorrelations over time were significant for both variables, indicating that the constructs were stable over the time period (see Figure 3). In our model, PNE at *t*1 is not a predictor of resilience at *t*2 but resilience at *t*1 is a significant negative predictor for PNE at *t*2, so hypothesis 2 is rejected (see Figure 3).

We tested RQ3 using model 3. Bivariate cross-sectional correlations between PNE and stress were significant at both measurement times (see Table 2). Likewise, the autocorrelations over time were significant for both variables, indicating that the constructs were stable over the time period (see Figure 3). For R1, resilience at *t*1 was a significant negative predictor for stress at *t*2 (see Figure 3).

## Discussion

### Discussion part 1

The aim of this study was to get deeper insights into PNE behavior and causal associations of PNE with perceived stress and resilience. We found that important reasons for PNE intake are general performance pressure, cognitive performance pressure and long working hours from an expert judgement. PNE with soft enhancers is widespread among students in Germany, while at the same time the use of prescription/illicit substances is not commonplace according to students' self-reports. We found that perceived stress predicted a higher use of PNE and that resilience predicted lower use of PNE. At the same time, PNE predicted higher levels of stress.

In part 1 of our study, OPs' estimate of the prevalence rate was in the middle range of those reported in the literature: At 10.9%, it is slightly higher than the prevalence rate of 8.4% reported by Müller et al. (21), who conducted a survey on PNE_PD_ use among German employees. This difference may be explained by the social desirability bias: The self-reported prevalence rates of employees might be lower than those raised by indirect query, because employees might consider the use of PNE as socially not acceptable. In comparison to their assumption of 10.9% of the employees using PNE_PD_, a survey conducted regularly by one of the biggest German health insurances showed that only 2% of German employees use PNE_PD_ on a regular basis and reported a lifetime-prevalence rate of 5.5% (57).

General and cognitive performance pressure were the most rated reasons for PNE_PD_ use by the asked OPs in part 1 of the study. According to prior research, improving cognitive performance is one of the main motives for PNE_PD_ (15). In 2015, Schröder et al. (22) reported that one reason for the rather low prevalence rates among employees might be that they only use PNE_PD_ selectively when the perceived burden of meeting their jobs demands is high. In future studies, it should therefore be precisely recorded in which period of time PNE was used. Both the duration and regularity of PNE used are key for the health consequences of PNE and in relation to stress at the workplace. Also, the relationship between PNE use at work and recreational use, and thus the relationship between PNE and addiction to medicine should be further investigated in future studies, because the use of prescription and illicit drugs overlap for recreational use and PNE.

It is a strength of part 1 of the study that OPs were interviewed and thus the potential for a social desirability effect was reduced (46). Apart from that, OPs may have valuable insights on the role PNE_PD_ plays for the employees they supervise, since they are direct contacts for employees of a respective organization in health matters. On the other hand, the OPs' view could be distorted through the glasses of their occupation. It is possible that the OPs' attention is more focused on workers whose stress levels they consider high and who they know are using PNE_PD_. This could lead to a bias in the assessment of prevalence rates. In addition, there is a selection bias with respect to the OPs' employees. It is likely, for one, that OPs are frequented primarily by employees at medium or large companies. Small companies and the self-employed may be underrepresented accordingly. Furthermore, at-risk employees that suffer from high levels of performance pressure are more likely to consult their OP.

### Discussion part 2

In part 2 of our study, we asked students about their PNE behavior. This increased the risk of social desirability bias but also allowed for a more fine-grained insight into PNE behavior in this at-risk population. 83.1% of part 2 participants believed that prescription/illicit substances are used without medical indication to improve cognitive functions. This observation is in line with the study by Middendorff et al. (2) in which 84% of respondents had heard of the possibility of taking substances to enhance cognitive performance. Although it is not possible to draw a direct conclusion from the level of awareness of PNE to prevalence rates of PNE, it is possibly rather the high level of awareness that gives rise to a continued discussion on PNE than the actual prevalence rates of PNE.

We also showed in part 2 that 76.8% of the students had used soft enhancers in the last month and 5.4% had used prescription/illicit drugs for PNE in the last month. Until now, little is known about the percentages of German students using soft enhancers as PNE. Middendorff et al. (2) reported a prevalence rate of 5% of students using soft enhancers. However, their result is hardly comparable to our findings due to differential operationalizations: While e.g., drinking coffee for improving cognitive functions did not count as soft enhancement in the study by Middendorff et al. (2), in our study this was explicitly included in the operationalization of soft enhancers. Franke et al. (58) reported that 88.1% of German students and alumni used any soft enhancer, including e.g., coffee. Their results are in line with our results because of a similar definition of soft enhancers. Although the percentage of prescription/illicit substance users is much lower than the percentage of soft enhancers in part 2 of our study, it is still high compared to other last-months prevalence rates reported in the literature: In a sample of 5.882 German university students, Sattler et al. (19) reported that 1.15% of their respondents had engaged in PNE during the previous month. A possible reason for the higher last-month prevalence rate in our study may be that in our definition of PNE we included the use of cannabis for compensation of study stress, which was the most reported substance for PNE. This is supported by data published by Dietz et al. (59) who report 12-month prevalence rates between 8 and 11.3% for German university students and state that cannabis was the most used substance for PNE. The prevalence rates for the substances typically thought of when discussing PNE, such as methylphenidate, modafinil and other amphetamines, show prevalence rates that are closer to the results of Sattler et al. (19). Another indicator that last-month prevalence rates are highly dependent on the definition used of PNE is shown in comparison to the results of the cross-sectional study conducted by Bagusat et al. (36) who found a last-month prevalence rate of 10.1% for prescription/illicit substances. However, the authors included mood enhancing in their definition of PNE, which we regarded as a recreational use of prescription/illicit substances in our study and therefore excluded from our definition. Maier and Schaub (9) proposed that students report PNE particularly during exam periods. This is also shown by our results, as there is a significantly higher last month prevalence rate for students preparing for exams during the last month than for students not preparing for exams.

The results on prescription/illicit substances of part 2 in the present study must be regarded with care, because prevalence rates and therefore total numbers of users are low and our sample cannot be considered to be representative. Maier and Schaub (9) argue that high prevalence rates reported in American studies may at least be partially because of the inclusion of prescription/illicit drug use that failed to distinguish between recreational use and PNE within academic contexts. The fact that the recreational use of prescription/illicit substances makes a considerable share in the overall use of these substances is also supported by our data, as the most prevalent reasons for taking illicit/prescription substances mentioned in our survey were recreational purposes. The findings from the qualitative interviews by Hildt (60) also support that conclusion and suggest that prescription/illicit substances are not used in an academic context in first place but are initially used to have time and energy for leisure activities. These substances in turn are used for PNE in an academic context when users discover properties of the substances that could also be used for exam preparation. Since these individual case reports roughly give a conclusive picture with our data, this hypothesis should be investigated in further quantitative research with several measurement points. The use of cannabis to compensate for study stress was the most important single factor concerning prescription/illicit substances in our study. Further studies could therefore investigate whether the use of cannabis to compensate for study stress is more likely to be preceded or followed by use for recreational purposes.

### Discussion part 3

In the final part 3 of our study, we assessed PNE, stress and resilience in a longitudinal study design to get deeper insights about the time-lagged directed correlation between the study variables. H1 in part 3 was accepted. Therefore, stress might cause a higher level of PNE, but PNE also causes stress to a lesser extent. Still, both effects are small as well as the difference between both effects. The finding of a small effect of stress on PNE is in line with Schröder et al. (22), who reported that PNE is only used in extremely stressful situations. In those situations, PNE is used to cope with excessive workload, but not as a long-term strategy to gain an advantage. Although the results of Schröder et al. (22) refer to the use of prescription/illicit substances, this consideration could also apply to soft enhancers because, as we were able to show in part 2 of our study, these are also consumed primarily during times of very high workload, such as exam preparation time. Furthermore, the study by Jebrini et al. (20), who conducted a survey among medical students in Germany on PNE, stress and resilience, and also included soft enhancers in their definition of PNE, shows that PNE seems to be associated with stressful situations. This supports our findings as well as the results of a study conducted by Wolff et al. (61) who integrated the use of PNE in a higher education context into the Job Demands Resources Theory: they reported that the intake of prescription drugs is associated with higher burnout scores among students. Furthermore, PNE might result in increased strain and therefore decreased cognitive performance. Although the effect of stress on PNE was small in our study, our results, taken together with those of Wolff et al. (61), could suggest that not only does high workload and performance pressure lead to PNE, but PNE in turn influences how stress is perceived and might therefore impair health in terms of strain outcomes.

For model 2 in part 3 of our study we hypothesized that there is a negative cross-lagged association between PNE and resilience. In our model, PNE at *t*1 was a not predictive of resilience at *t*2, but resilience at *t*1 was a protective factor for reduced use of PNE at *t*2. Therefore, resilience might cause a lower use of PNE. This result is supported by Bagusat et al. (36) who reported that subjects who are less resilient had a higher risk of using PNE. Our data is furthermore supported by the study of Jebrini et al. (20) who also used the BRS in their study for assessing resilience and showed that there was an association between PNE use and lower scores in the BRS. Still, their data is not comparable to ours altogether, because the surveyed substances were different. In comparison to that, Chmitorz et al. (27) could not show an effect of resilience on PNE in a sample of spine surgeons. This may be explained by the different study sample or other differences in the study methods such as the conceptualization of PNE. That the effect of resilience on PNE in our study is rather small might be explained by other influencing factors already discussed in part 2 of our study, such as the recreational use of psychoactive substances.

Recall that for model 3 we assumed a negative association between resilience at *t*1 and stress at *t*2. Resilience at *t*1 was a significant negative predictor for stress at *t*2. We can therefore assume that a higher level of resilience is a protective factor in stress prevention. A significant causal association between stress at *t*1 and resilience at *t*2 was not shown, so stress itself seems not to have an influence on resilience. From a theoretical point of view this makes sense, because only positive adaptations to stressful events may lead to resilience (42). But when resilience proceeds stressful periods of time, as e.g., exam preparation, individuals might already have adaptive coping styles to perceive less strain. This interpretation is also supported by Lupe et al. (45), who suggest that developing resilience may lead to less perceived stress.

Summarizing part 3 of our study, we showed that not only stress has an influence on PNE, but also PNE could cause stress, and that resilience is a protective factor against perceived stress and the use of PNE. A strength of the third part of the study is the longitudinal study design. Especially when conceptualizing resilience as a dynamic process of adaptation in the face of adversity, it will not be possible to predict resilience by a single baseline measurement (50). Therefore, Kalisch et al. (51) as well as Kunzler et al. (42) underline the need for longitudinal prospective studies on resilience. At the same time, it is a weakness of the study that it has only two measurement points and not several to investigate the causalities between PNE stress and resilience more in depth. Another weakness is measuring resilience with the BRS. Although the BRS is the best approximation to the concept according to Windle et al. (49), an approximation to the broad discourse on the construct resilience as presented in the introduction is hardly possible by a questionnaire. For a more integrative understanding of the causal associations between PNE stress and resilience, further influencing factors like habits or subjective norms should be considered in future longitudinal studies.

### General discussion and conclusion

Taking together the results of the different parts of the study the difference between the OPs presumed prevalence rate of PNE in study 1 and the actual lower prevalence rate among students from part 2 is striking, considering that students are more likely to use PNE than people in employment (9). Scientific literature agrees that the use of PNE is rather low in the general population (23). What should rather be alarming is that overestimations of prevalence rates and also about the effectiveness of PNE could eventually lead to more PNE use among young people, since they might consider the use of psychoactive substances for enhancing their cognitive performance the norm (62). This is also supported by the data of a big German health insurance, which shows that the prevalence rate of PNE has stayed the same since a few years in Germany, but more and more people know about PNE as a possibility to enhance cognitive performance (58). An informal survey conducted by Maher in 2008 showed that 69% of participants would even risk mild side effects for taking PNE (7). This is also supported by our data since most of the OPs think that the use of PNE is increasing (part 1) and 83.1% of the students believed that prescription/illicit substances are used without medical indication to improve cognitive functions (part 2). Although comparisons between the different parts of the study must be handled with care due to the different methodological approaches, an ongoing public discussion about PNE is needed (6). Future studies should investigate to what extent PNE during studies can be a predictor for later stress consequences such as addiction disorders or other mental illnesses.

The main reasons for PNE intake suspected by the OPs (part 1) can to some extent be transferred to an academic context. The OPs suspected performance pressure and long working hours to be the main reasons for PNE use. In part 2 of our study, we showed that improving cognitive functions and compensation of study stress are important reasons for substance use. The wish to improve cognitive functions or compensate for study stress may origin from performance pressure, long working hours, or an extensive workload. Therefore, the assumptions made by the OPs about the reasons for PNE also represent the reality of students in an academic context quite well. This is further supported by the longitudinal results of our study, where we showed that PNE and perceived study stress are interrelated, and that resilience can influence both perceived study stress and PNE use in terms of an adaptive coping style. Although the effects in part 3 of our study were small, the results make sense when considering Resilience Theory: our data support the theoretical assumptions that a positive adaptation to stressors fosters resilience, protecting us against maladaptive coping strategies such as PNE (32, 39).

The present study has three notable strengths. Firstly, in part 2 of our study we investigated the prevalence rate including soft enhancers. This is important on the one hand, because little is known about how many students in Germany use e.g., caffeine for improvement of cognitive functions. On the other hand, this could be an important contribution toward shifting the debate about PNE. As Franke and Lieb (6) argued, caffeine can be considered a good alternative to prescription/illicit substances for PNE, as, in contrast to e.g., methylphenidate or modafinil, the safety and effectiveness of caffeine is assured (63). Secondly, all our studies offer a clear definition of PNE and therefore an explicit distinction between recreational use of psychoactive substances and PNE. This could contribute to better understanding the dynamics in substance use inside and outside of an academic context in the future. Thirdly, it is a strength of our study that for prevalence rates we either used expert ratings from OPs or an anonymous online questionnaire for all study parts thereby reducing the social desirability effect (46). The different methodological approaches therefore offer a more differentiated picture of PNE use in at-risk populations.

At the same time, this study has several limitations. Firstly, although the last-month prevalence rate in the month prior to the exams seems to be an acceptable marker of current PNE use (64) one last-month prevalence rate is not enough to draw a conclusive picture of PNE use in an academic context in general. More studies with a longitudinal study design and more observation points are needed to get an even better insight into PNE consumption behavior. Secondly, the self-selection process used in our survey could lead to underestimating or overestimating the use of PNE. For lack of brightfield data, we did a darkfield investigation on self-reported PNE use among students in the main part of the study. However, because self-reports may be biased due to social desirability, part 1 of our study first asked experts to provide information on the same question. Their estimates of prevalence are also imprecise because they may only learn about the more difficult cases in need of treatment. However, both perspectives to get an approximation toward the actual prevalence rates. At the same time, the different methodological approaches, especially the different survey technique and the different definitions of PNE, make it harder to compare the results of part 1 to the results of part 2 and 3. Thirdly, we did not consider alcohol as a source for PNE in this study. The use of alcohol for PNE is in many ways special and distinct from other substances used for PNE (9). Therefore, the use of alcohol for PNE should be considered in a separate investigation. Because of the longitudinal study design in part 3 of our study, we can get a glimpse of what causal relations between PNE, stress and resilience could look like. But two measurements with a time lag of 1 month are not enough to get a final insight on the causal interactions of PNE, stress and resilience. Therefore, future studies could focus on conducting surveys with more than two time-lagged measurements.

Performance pressure, a heavy workload and stress are an everyday reality for many students and employees. According to our results, resilience is a relevant factor in averting dysfunctional coping strategies for study stress. Intervention and prevention strategies should therefore focus on promoting resilience and preventing maladaptive responses, such as the use of PNE, to perceived stress.

## Data availability statement

The raw data supporting the conclusions of this article will be made available by the authors, without undue reservation.

## Ethics statement

The studies involving human participants were reviewed and approved by Die unabhängige Ethik-Kommission, Medical Faculty of RWTH Aachen University (EK 412-19). The patients/participants provided their written informed consent to participate in this study.

## Author contributions

Conceptualization, investigation, and project administration: PG, JL, and JD. Data curation: PG, RP, JD, and LN. Formal analysis and software: PG, RP, and JD. Methodology and writing-review and editing: PG, JL, JD, and RP. Supervision: JL and PG. Validation: PG, JL, LN, RP, and JD. Visualization and writing-original draft preparation: JD. All authors agree to be accountable for the content of the work.

## Conflict of interest

The authors declare that the research was conducted in the absence of any commercial or financial relationships that could be construed as a potential conflict of interest.

## Publisher's note

All claims expressed in this article are solely those of the authors and do not necessarily represent those of their affiliated organizations, or those of the publisher, the editors and the reviewers. Any product that may be evaluated in this article, or claim that may be made by its manufacturer, is not guaranteed or endorsed by the publisher.
