# Supplementary material for: The relevance of pharmacological neuroenhancement for stress and resilience—A multistudy report
Source: Front Public Health. 2022 Nov 11;10:971308. doi: 10.3389/fpubh.2022.971308 (PMC9692085; doi:10.3389/fpubh.2022.971308)
Supplement: Supplementary file 1 [file Table_1.pdf]

Supplement table 1. Sociodemographic and job characteristics of the surveyed occupational physicians (OPs)

	n	%
**Gender**	**152**	
Female	78	51.3
Male	74	48.7
**Professional Background**	**149**	
Specialist in occupational medicine	104	69.8
Additional designation occupational medicine	29	19.5
Resident in training	16	10.7
**Years of professional experience as OPs**	**148**	
1-5 years	32	21.6
6-10 years	12	8.1
11-20 years	55	37.2
21-30 years	38	25.7
31-40 years	11	7.4
**Type of employment**	**149**	
internal/employed	68	45.6
External/freelance	43	28.9
Intercompany service	38	25.5

The final sample consisted of 152 OPs. n = number of OPs, % = percent
