# Supplementary figures and images for: The relevance of pharmacological neuroenhancement for stress and resilience—A multistudy report
Source: Front Public Health. 2022 Nov 11;10:971308. doi: 10.3389/fpubh.2022.971308 (PMC9692085; doi:10.3389/fpubh.2022.971308)

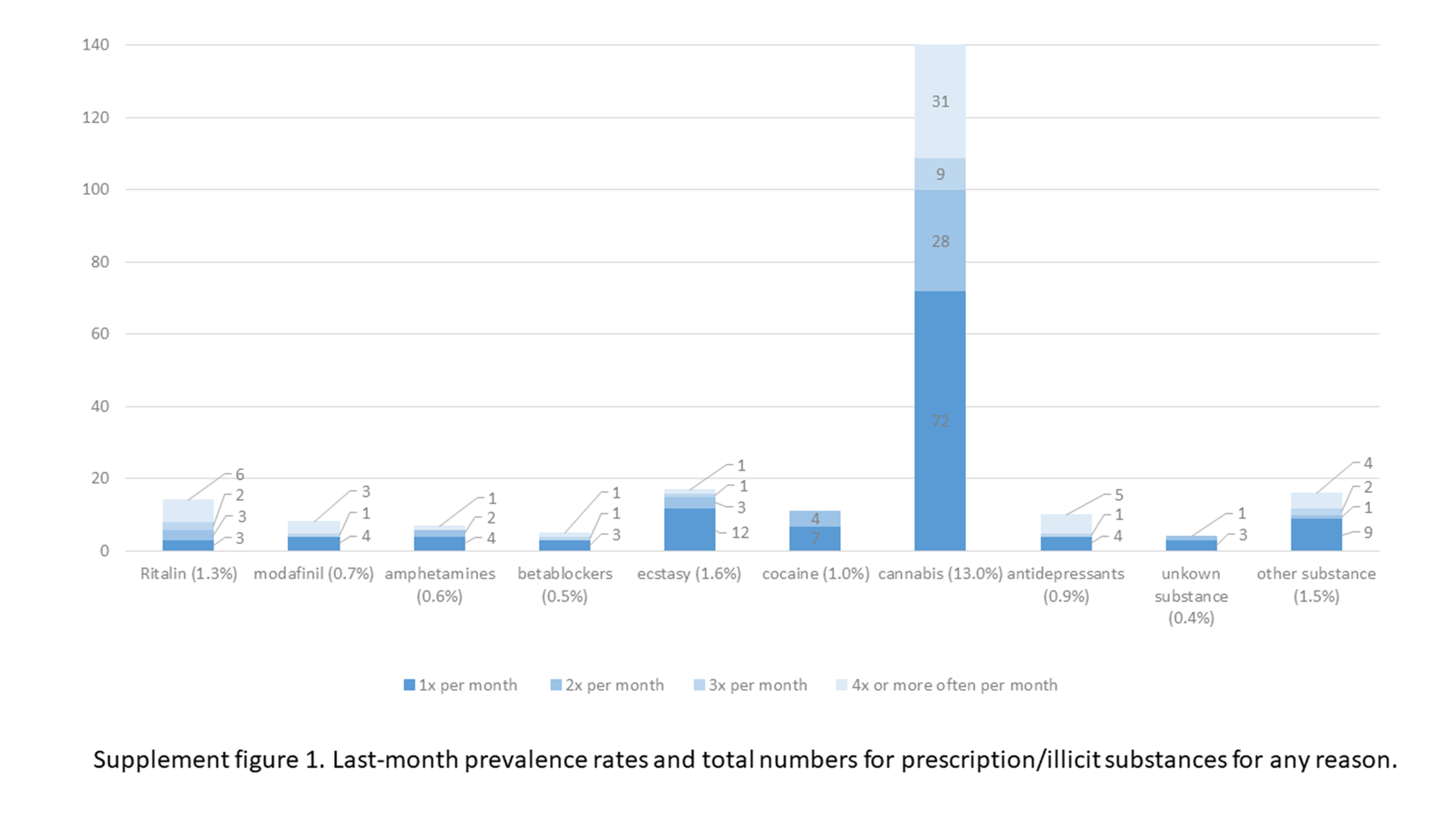

Supplement: Supplementary file 2 [file Image_1.TIF]
